# Identification of differentially expressed genes and pathways in BEAS-2B cells upon long-term exposure to particulate matter (PM_2.5_) from biomass combustion using bioinformatics analysis

**DOI:** 10.1265/ehpm.22-00272

**Published:** 2023-09-15

**Authors:** Qian Yuan, Haiqiao Zhang

**Affiliations:** Dongguan Maternal and Child Health Care Hospital, Dongguan, 523120, China

**Keywords:** PM_2.5_, KEGG, Protein-protein interaction, Immune cell infiltration

## Abstract

**Background:**

Long-term exposure to PM_2.5_ from burning domestic substances has been linked to an increased risk of lung disease, but the underlying mechanisms are unclear. This study is to explore the hub genes and pathways involved in PM_2.5_ toxicity in human bronchial epithelial BEAS-2B cells.

**Methods:**

The GSE158954 dataset is downloaded from the GEO database. Differentially expressed genes (DEGs) were screened using the limma package in RStudio (version 4.2.1). In addition, DEGs analysis was performed by Gene Ontology (GO) functional analysis and Kyoto Encyclopedia of Genes and Genomes (KEGG) pathway analysis. A protein-protein interaction (PPI) network was constructed, MCODE plug-in and the cytoHubba plug-in in Cytoscape software was used to identify the hub genes. Finally, CytoHubba and DEGs were used to integrate the hub genes, and preliminary validation was performed by comparing the toxicology genomics database (CTD). Differential immune cell infiltration was investigated using the CIBERSORT algorithm.

**Results:**

A total of 135 DEGs were identified, of which 57 were up-regulated and 78 were down-regulated. Functional enrichment analyses in the GO and KEGG indicated the potential involvement of DEGs was mainly enriched in the regulation of endopeptidase activity and influenza A. Gene Set Enrichment Analysis revealed that Chemical Carcinogenesis - DNA adducts were remarkably enriched in PM_2.5_ groups. 53 nodes and 198 edges composed the PPI network. Besides, 5 direct-acting genes were filtered at the intersection of cytohubba plug-in, MCODE plug-in and CTD database. There is a decreasing trend of dendritic cells resting after BEAS-2B cells long-term exposure to PM_2.5_.

**Conclusions:**

The identified DEGs, modules, pathways, and hub genes provide clues and shed light on the potential molecular mechanisms of BEAS-2B cells upon long-term exposure to PM_2.5_.

**Supplementary information:**

The online version contains supplementary material available at https://doi.org/10.1265/ehpm.22-00272.

## 1. Introduction

Particulate matter 2.5 (PM_2.5_) refers to particulate matter with an aerodynamic diameter ≤2.5 µm in the atmosphere [1]. PM_2.5_ is the most common component of air pollution in developing countries and poses a significant threat to human health [2]. Residential biomass combustion is also reported to be one of the major sources of PM emissions [3]. Epidemiological studies have demonstrated that long-term exposure to airborne PM_2.5_ is closely related to the increased prevalence and mortality of human respiratory and cardiovascular diseases [4, 5]. Early PM_2.5_ exposure levels may affect lung development and have a potential impact on the disease burden of respiratory diseases associated with ambient PM_2.5_ exposure later in life [6]. Besides, the molecular toxicity mechanism of PM_2.5_ is connected to oxidative stress response, inflammatory response, genotoxicity and immunotoxicity [7, 8]. Therefore, it is critical to investigate the potential toxicological mechanism of PM_2.5_ and respiratory system injury.

Long-term exposure to PM_2.5_ from biomass combustion has previously been shown to increase intracellular reactive oxygen species (ROS) levels and susceptibility to microbial infections and other lung diseases in bronchial epithelial BEAS-2B cells [9]. Although PM_2.5_ has been proven to be toxic to humans, its mechanism of action remains unclear. This may be due to the limitations of traditional studies, which have focused too much on single genes or pathways and ignored the relationships between genes [10]. In recent years, with the development of next-generation sequencing technology, high-throughput screening of toxicological relationships has been achieved through genomic analysis of bioinformatics, rapid mapping of biological pathways and genes involved in toxicological interference, and effective analysis of mechanisms and pathways. The emergence of high-throughput sequencing technology has become an effective way to elucidate the pathogenic genes of a variety of human diseases, which are helpful to explore the potential mechanism of toxicity [11, 12]. For example, a study by Cai et al. using the Gene Expression Omnibus (GEO) database identified nine hub genes of Polybrominated diphenyl ether that may play an important role in their toxicological mechanism [13].

In this study, we re-analyzed the gene expression profile of GSE158954. Gene Ontology (GO) and Genes and Genomes (KEGG) pathway enrichment analyses were performed for Differentially expressed genes (DEGs). The hub genes in the key modules were identified and simply validated against the comparing the toxicology genomics database (CTD). The findings of hub genes and pathways in BEAS-2B cells after long-term exposure to PM_2.5_ from biomass combustion provide a new research direction.

## 2. Methods and materials

### 2.1 Data source

The dataset was obtained from the Gene Expression Omnibus database (GEO, https://www.ncbi.nlm.nih.gov/) in NCBI based on the keywords “PM_2.5_” and “BEAS-2B cells” and “Biomass Combustion” and “Homo sapiens”. The dataset GSE158954 meets the retrieval requirements. The data set GSE158954 was based on the GPL570 platform, including 6 samples, 3 from PM_2.5_ groups and 3 from controls. The PM_2.5_ in the GSE158954 dataset in this paper was obtained from biomass combustion. Briefly, pellets for biomass combustion were obtained from a biomass power plant (Bürger Energie St. Peter eG, St. Peter, Schwarzwald, Germany), which burns only softwood chips (mainly spruce) from local forests. The wood chips consist of barked stem wood and branches of at least 7 cm in diameter (marketable wood), free of leaves and twigs. The maximum burning temperature is 910 °C. The collected bulk fly ash was size fractionated by a cyclone, where particles with an aerodynamic cut-off diameter of 2.5 µm were used for bioassay. The BEAS-2B cells were treated with 100 µg/mL of PM_2.5_ for 5 weeks to observe the changes in gene expression induced by PM_2.5_ exposure. Gene expression of BEAS-2B cells long-term exposed to PM_2.5_ was analyzed using microarray technology [9].

### 2.2 Identification of differentially expressed genes

Use of statistical software RStudio (Version 4.2.1, https://rstudio.com/) and Bioconductor packages (http://www.bioconductor.org/) for bioinformatics analysis of PM_2.5_ groups and the control groups. After downloading the matrix data, the quantile normalization method was used to standardize the gene expression matrix between groups. The limma package was used to explore the significant differentially expressed RNAs. Heatmap package (http://www.bioconductor.org/packages/release/bioc/html/heatmaps.html) was installed to produce the heatmap. The adjusted *p* values <0.05 and |log FC| > 1 were considered statistically significant.

### 2.3 Functional enrichment analyses of the DEGs

Firstly, the org.Hs.eg.db package was used to convert the name of the interested gene into a gene ids recognized by RStudio. GO consists of 3 major components, including biological processes (BP), cell components (CC), and molecular functions (MF), which was to explore the biological significance of genes of interest. KEGG enrichment analysis was performed on the genes of interest to identify the key pathways closely related to the occurrence and development. The adjusted *p* < 0.05 was considered statistically significant.

### 2.4 Gene set enrichment analysis

Gene Set Enrichment Analysis (GSEA) was used to detect whether the enrichment of KEGG pathways in PM_2.5_ and control groups was statistically significant. False discovery rate (FDR) <0.25 and *p* < 0.05 were regarded as the cutoff criteria.

### 2.5 Identification of hub genes in the regulation network

The protein-protein interaction (PPI) network of key modules was constructed using the STRING online database (https://string-db.org/) [14], by searching limitation of “Homo sapiens” and a score >0.7 in accord with high confidence interaction as significant value, and the unconnected nodes in the network were hidden. Cytoscape software (version 3.8.2) [15] was used to visualize the PPI network. Further, meaningful modules in the PPI network were obtained by MCODE plug-in [16], with degree cutoff = 2, maximum depth = 100, and k-score = 2. Hub genes were identified using the cytohubba plug-in [17] in Cytoscape software.

### 2.6 Immune cell infiltration analysis

The CIBERSORT algorithm was used to analyze the proportion of 22 immune cells in the samples. The samples with *p* < 0.05 were significant [18]. Pearson correlation analysis was performed for the correlation coefficients between the 22 immune cells. We then investigated the difference in immune cell infiltration between the exposed PM_2.5_ groups and the control groups. Pearson correlation analysis was used to calculate the correlation coefficient between the infiltration of different immune cells and core genes.

## 3. Result

### 3.1 Identification of differentially expressed genes

**Fig. 1:**
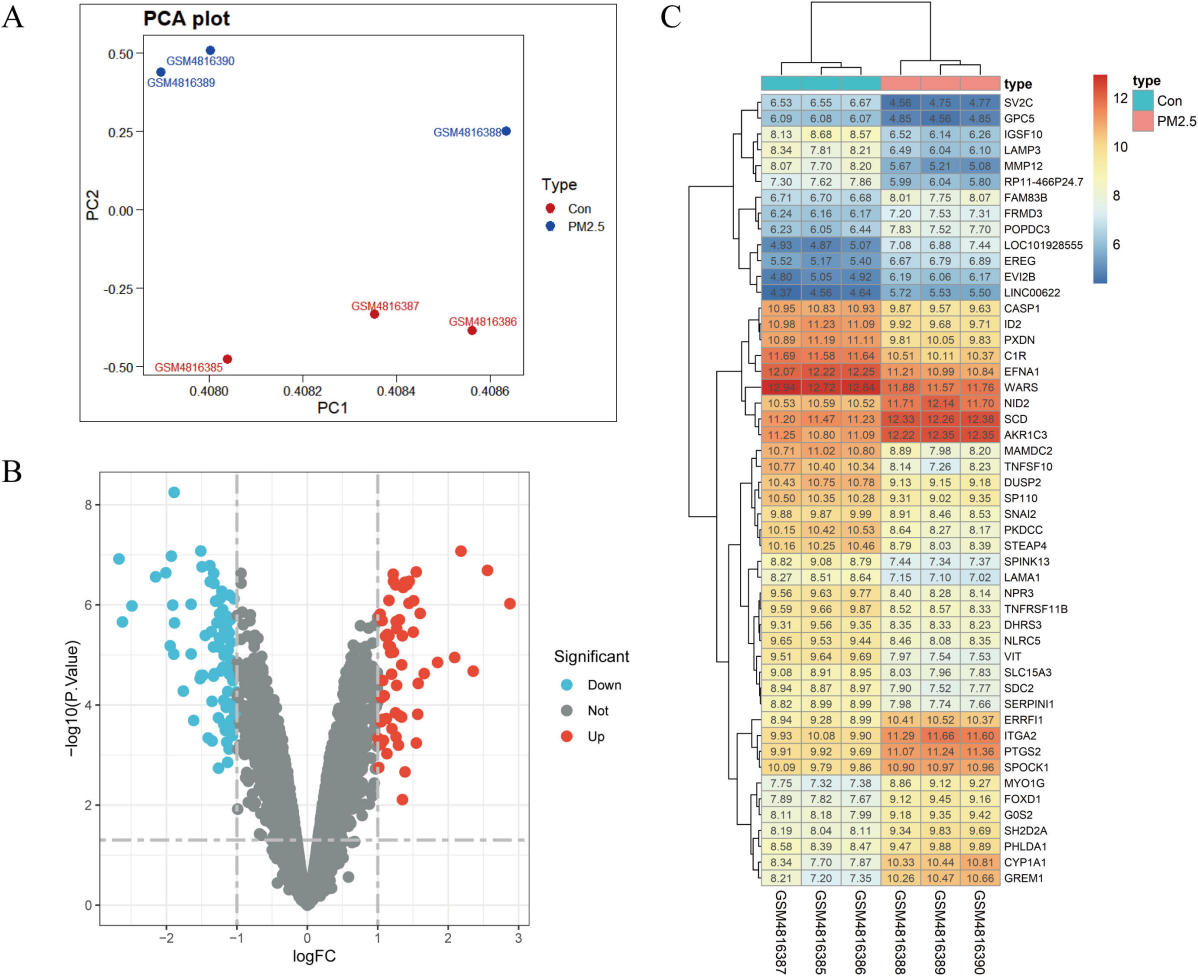
DEGs screening between PM_2.5_ groups and control groups. (A) Principal component analysis. Red dots are the control groups and blue dots are the PM_2.5_ groups. (B) Volcano plot of differentially expressed genes. Red dots indicate up-regulated DEGs and green dots indicate down-regulated DEGs. (C) Heat map visualization samples of the top 50 DEGs between PM_2.5_ groups and control groups.

The Microarray datasets GSE158954 were standardized, and the results are shown in Supplementary Fig. 1S. PCA data showed that the expression of genes was different between PM_2.5_ groups and control groups (Fig. 1A). When the dataset was screened by the limma package (corrected P-value <0.05, log FC > 1), 135 DEGs were obtained. Among them, 57 up-regulated genes and 78 down-regulated genes were identified in PM_2.5_ group (Fig. 1B). The cluster heatmaps of the top 50 DEGs are shown in Fig. 1C.

### 3.2 GO term enrichment and KEGG pathway analysis of DEGs

**Fig. 2:**
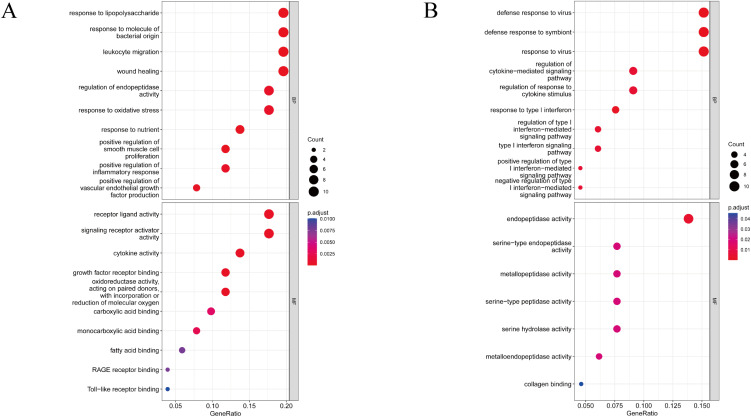
The GO analyses for significant DEGs. Bubble plots show the results of GO analysis of (A) up-regulated DEGs and (B) down-regulated DEGs.

**Fig. 3:**
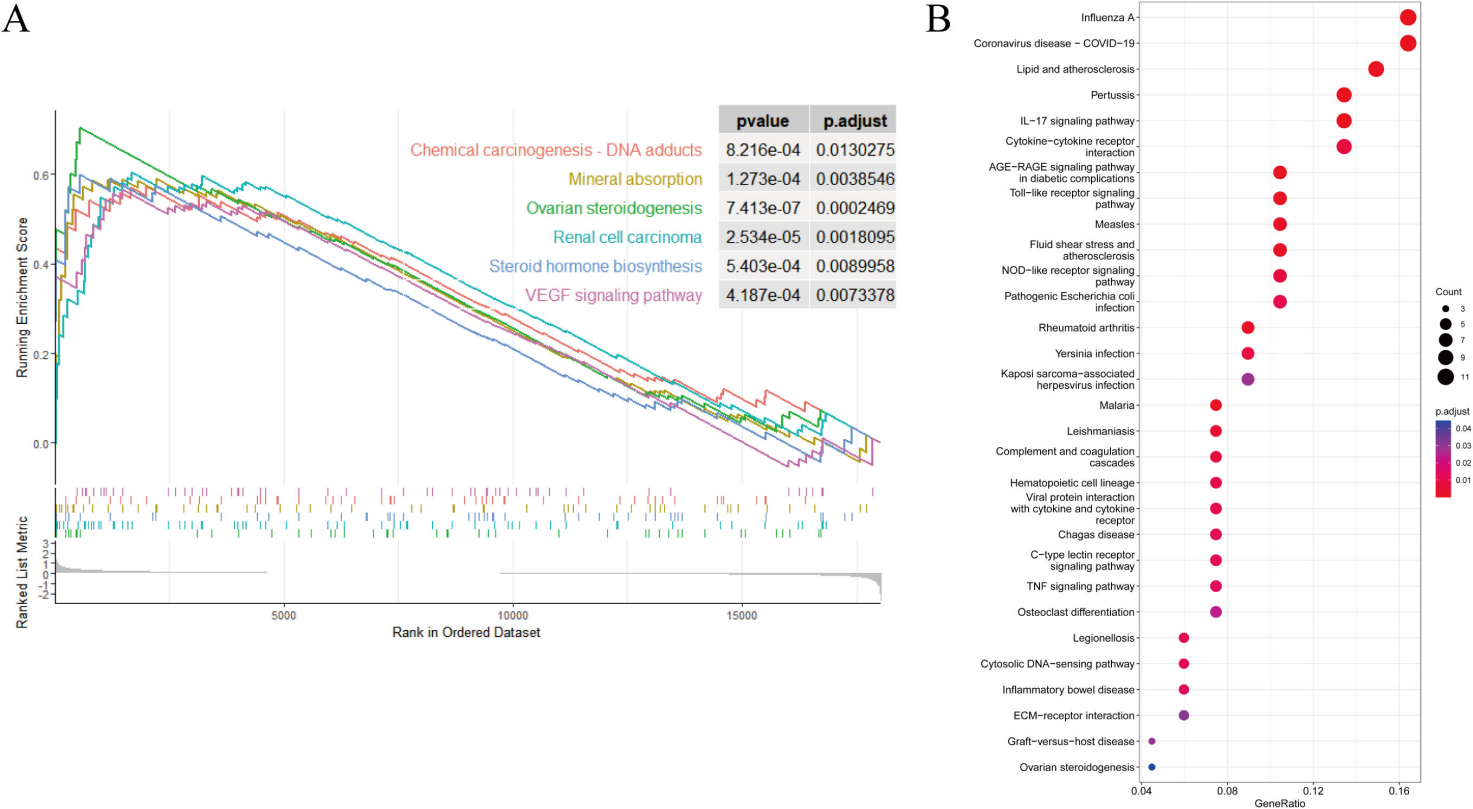
GSEA and KEEG analysis. (A) Gene set enrichment analysis. (B) The KEGG enrichment analyses for significant DEGs.

The significant up-regulated and down-regulated DEGs were utilized for GO analyses. GO functional enrichments of up-regulated and down-regulated genes with a p-value of <0.05 were obtained. The results are shown in Fig. 2 and Supplementary Fig. 1S. The BP of the up-regulated DEGs were mainly involved in response to lipopolysaccharide, response to molecule of bacterial origin, leukocyte migration, response to nutrient, wound healing, positive regulation of smooth muscle cell proliferation, positive regulation of vascular endothelial growth factor production, regulation of endopeptidase activity and response to oxidative stress. The MF terms of the up-regulated DEGs mainly included growth factor receptor binding, cytokine activity, receptor ligand activity, signaling receptor activator activity, oxidoreductase activity, acting on paired donors, with incorporation or reduction of molecular oxygen, monocarboxylic acid binding, carboxylic acid binding and cytokine receptor binding (Fig. 2A). The BP of the down-regulated DEGs mainly included defense response to the virus, defense response to symbiont, response to virus, regulation of cytokine–mediated signaling pathway, regulation of response to cytokine stimulus, response to type I interferon and regulation of type I interferon–mediated signaling pathway. The MF of the down-regulated DEGs mainly included endopeptidase activity, serine-type endopeptidase activity, metallopeptidase activity, serine-type peptidase activity, serine hydrolase activity, metalloendopeptidase activity and collagen binding (Fig. 2B). The enriched KEGG pathways mainly included Pertussis, IL-17 signaling pathway, Influenza A, Coronavirus disease - COVID-19, Lipid and atherosclerosis, AGE-RAGE signaling pathway in diabetic complications, Toll-like receptor signaling pathway, Cytokine-cytokine receptor interaction and MAPK signaling pathway (Fig. 3B).

### 3.3 GSEA analysis

The distribution of the pathway gene sets on all gene expression data from the PM_2.5_ groups and controls were explored using the R package. Results revealed that the pathways of Chemical carcinogenesis - DNA adducts, Mineral absorption, Ovarian steroidogenesis, Renal cell carcinoma, Steroid hormone biosynthesis and VEGF signaling pathway were enriched in PM_2.5_ groups (Fig. 3A).

### 3.4 PPI network analysis of DEGs

**Fig. 4:**
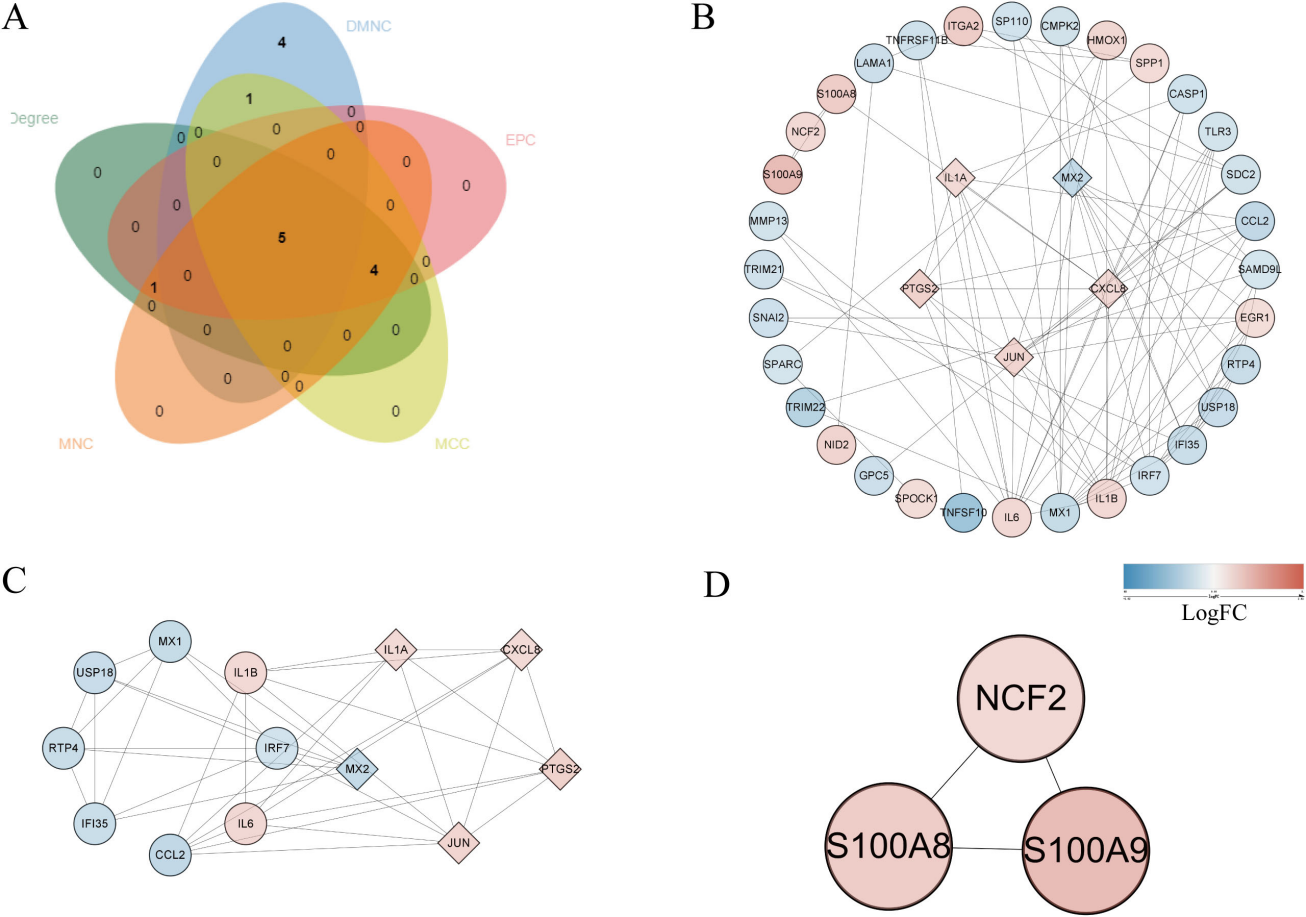
The PPI network and venn plot of hub genes. (A) The overlapped hub genes from different algorithms. (B) The PPI network for the 5 hub genes consisted of 37 nodes and 91 edges. (C) and (D) are module 1 and module 2, respectively. The color of the node indicates the log FC of the central gene. The diamond represents the hub gene.

The 135 DEGs were imported into the STRING database (http://string-db.org) to construct the protein interaction network. The network diagram contains 53 nodes and 198 edges (Supplementary Fig. 2S). There were 22 up-regulated genes and 31 down-regulated genes in PPI network. In cytohubba plug-in, the top 10 hub genes were obtained by five algorithms: MCC, DMNC, MNC, Degree and EPC, as showed in Supplementary Table 1S. The overlapping hub genes in the five algorithms were analyzed by venn diagram, and finally five hub genes were identified, including JUN, CXCL8, MX2, IL1A and PTGS2 (Fig. 4A). The PPI network was composed of 37 nodes and 91 edges through Cytoscape (Fig. 4B). Using the MCODE plug-in in Cytoscape, two modules were identified. Module 1 (score = 6.167) contains 13 nodes and 37 edges (Fig. 4C), and module 2 (score = 3) contains 3 nodes and 3 edges (Fig. 4D). In addition, five hub genes retrieved by CTD were all associated with the toxic effects of PM_2.5_.

### 3.5 Immune cell infiltration

**Fig. 5:**
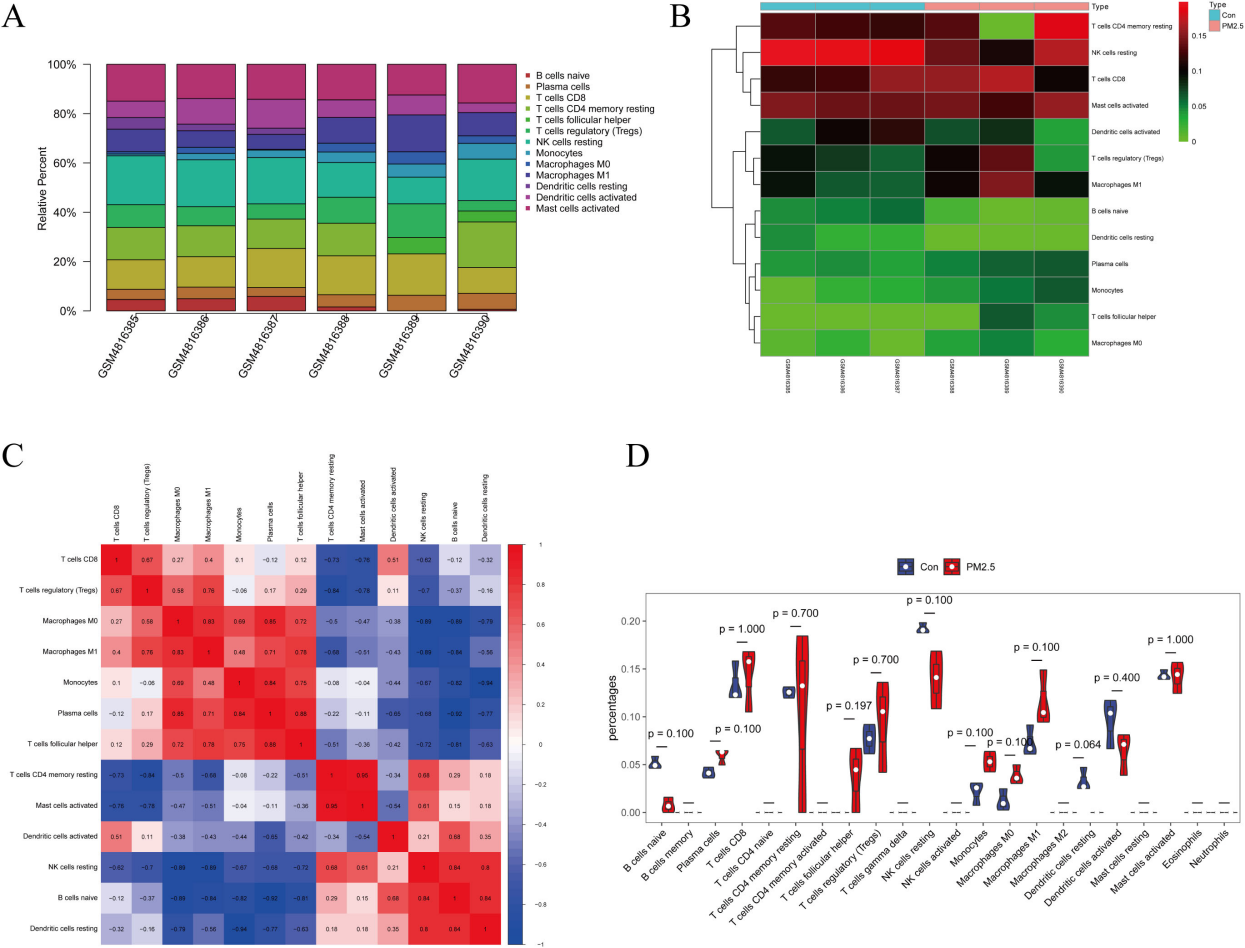
Immune cell infiltration in GSE158954. (A) The proportion of infiltrating immune cells in GSE158954. (B) The heatmaps of immune cells in control groups and PM_2.5_ groups. (C) Correlation matrix between immune cell types. Red means positive correlation, blue means negative correlation, and the darker the color, the stronger the correlation. (D) Variance analysis of immune cells in control groups (blue) and PM_2.5_ (red).

We obtained immune infiltration of 13 immune cell subgroups in the PM_2.5_ group and control group using the CIBERSORT algorithm. A bar plot and heatmap were generated to show the proportion of 13 immune cells in the 6 samples (Fig. 5A and Fig. 5B), and the co-expression correlation of various immune cells is displayed in Fig. 5C. Mast cells activated had the strongest positive correlation with T cells CD4 memory resting (correlation coefficient, 0.95), whereas Monocytes had the strongest negative correlation with Dendritic cells resting (correlation coefficient, 0.94). However, compared with control groups, although there was no statistical difference in the changes of 13 kinds of immune cells, the Dendritic cells resting showed a downward trend (Fig. 5D).

## 4. Discussion

With global population growth, urbanization, land desertification, and industrial pollution, the average concentration of PM_2.5_ is increasing worldwide, and the resulting environmental and health problems have attracted great attention [19]. PM_2.5_ was obtained from a biomass power plant. The main components included mineralogy and PAHs, as detailed in the supplementary Table 2S and Table 3S. Long-term PM_2.5_ exposure is significantly associated with increased mortality from respiratory disease, lung cancer, and cardiovascular disease [5]. In vitro studies confirmed the dysregulation of gene expression in BEAS-2B cells after PM_2.5_ treatment, leading to functional disorders, including oxidative damage, autophagy and apoptosis [20, 21]. Due to the complexity of molecular mechanisms in BEAS-2B cells caused by long-term exposure to PM_2.5_, the possibility of uncovering the mechanisms of gene-gene interactions requires the use of high-throughput methods to support classical toxicological analysis.

In this study, we analyzed the mRNA expression profile GSE158954 downloaded from the GEO database. The limma package was used to identify 135 DEGs between PM_2.5_ treated BEAS-2B cells group and the control group. The enriched BP terms were mainly response to lipopolysaccharide in the up-regulated genes. Lipopolysaccharide (LPS) can cause multiple organ damage. Severity and sensitivity of PM_2.5_ plus LPS to multiple organ damage in mice [22]. PM_2.5_-associated LPS is involved in the immune response of splenocytes to PM_2.5_ [23]. Simultaneous exposure of lipopolysaccharide (LPS) and urban particulate matter <2.5 µm (PM_2.5_) or desert dust exacerbated murine asthma [24]. PM_2.5_ also enhanced the lipopolysaccharide (LPS)-induced M1 polarization even though there was no evidence in the change of cell viability [25]. The enriched MF terms were the growth factor receptor binding in the up-regulated genes. Epidermal growth factor receptor (EGFR) is the most widely studied receptor tyrosine kinase. Environmental chemicals such as PM_2.5_ can also activate EGFR and become a risk factor for cancer [26]. The enriched BP terms were mainly defense response to virus in the down-regulated genes. Many epidemiological studies have reported the association between PM_2.5_ and influenza infection rates [27]. There is evidence that PM_10_ can inhibit the inflammatory response of alveolar macrophages under respiratory syncytial virus (RSV) infection, thereby promoting the spread of infection [28]. PM_2.5_ can reduce the expression of IL-6 and IFN-β in alveolar macrophages infected with influenza virus [29]. The enriched MF terms were the endopeptidase activity in the down-regulated genes. Peptidases play important roles in tumorigenesis, such as regulating bioactive peptides that are essential in tumor growth, degrading extracellular matrix, acting as adhesion molecules, or participating in intracellular signaling [30].

The enriched KEGG pathways showed that these 129 DEGs were significantly correlated with Pertussis, Influenza A, Coronavirus disease - COVID-19, Lipid and atherosclerosis, IL-17 signaling pathway. GSEA showed that the gene set Chemical carcinogenesis-DNA adducts were markedly enriched in the PM_2.5_ groups. It is associated with the incidence of tuberculosis and pertussis in heavily polluted areas of China [31]. Particulate matter pollution has been recognized as a risk factor for susceptibility and severity of coronavirus disease 2019 (COVID-19) and influenza viruses [32, 33]. Short-term exposure to PM_2.5_ significantly improved the survival rate of influenza A contaminated mice, while long-term inhalation of PM_2.5_ reduced the ability of lung macrophages to secrete IL-6 and IFN-β, resulting in lung tissue cell damage and down-regulating the immune defense mechanism of the lung [29]. Lipid deposition is one of the key factors contributing to the development of atherosclerosis. Epidemiological and animal experimental evidence suggests that PM_2.5_-induced atherosclerosis is mainly mediated through inflammation and changes in lipid metabolism [34]. A large number of studies have shown that PM_2.5_ can cause serum lipid metabolism disorders in ApoE−/− mice, promote the phagocytosis of ox-LDL by macrophages through surface scavenger receptors and induce foam cell formation [35–37]. PM_2.5_ inhibits autophagy in bronchial epithelial cells by activating phosphatidylinositol-3-kinase (PI3K)/protein kinase B (Akt) and mammalian target of rapamycin (mTOR) signaling pathways [38]. There was evidence of high dose nonlinearity and decreased DNA binding capacity in both leukocytes and lung cells from individuals exposed to high levels of PM compared with individuals exposed to low levels [39]. PM_2.5_ treatment changed the mRNA expression associated with the IL-17 signaling pathway in the lung and changed the mRNA expression associated with metabolic pathways in the liver [40]. However, IL-17 has been implicated in the occurrence and development of pertussis, COVID-19, influenza A, lipids and atherosclerosis [41–44]. Combined with our enrichment analysis results, we suggest that PM_2.5_ is largely associated with IL-17 signaling pathway.

In addition, five hub genes, JUN, CXCL8, MX2, IL1A and PTGS2 were determined based on the PPI network. To verify the expression of these genes in PM_2.5_, relevant literature was searched and summarized in Table 1. These genes need to be further verified by experiments, especially in PM_2.5_ [45–48]. The Jun (c-Jun, JunB and JunD) and Fos (c-Fos, FosB, Fra1 and Fra2) subfamilies are the major AP-1 proteins. Accumulating evidence suggests that AP-1 is involved in inflammation, differentiation, apoptosis, cell migration and wound healing [49]. PM_2.5_ exposure induces the activation of transcription factor AP-1 and its components c-Jun and ATF2 in human vascular endothelial cells, thereby promoting oxidative stress and inflammation [50]. PM_2.5_ up-regulates the expression of proline-rich small molecule protein 3 (SPRR3) by activating c-Jun transcription, and ectopic expression of SPRR3 inhibits ciliogenesis, causing epithelial damage and endothelial cell dysfunction [45]. The mRNA and protein expressions of c-fos and c-jun are increased, and CYP450s and GSTs are activated in the lung tissue of rats exposed to PM_2.5_, which is related to oxidative stress [51]. CXCL8 (Interleukin-8, IL-8) was originally described as a chemokine whose primary function is to attract infiltration of polymorphonuclear inflammatory leukocytes acting on CXCR1/2 [52]. IL-8 protein in the human airway is mainly produced by epithelial cells and plays an important role in mediating the pathogenesis of lung diseases caused by exogenous poisons. IL-8 is considered as a biomarker of airway inflammation caused by exogenous stimuli. Previous studies have shown that PM_2.5_ exposure can lead to excessive secretion of IL-8 protein by lung epithelial cells [53]. Air pollutants including crude PM_2.5_, extracted PM_2.5_ and Asian dust particles have been reported to stimulate the release of IL-6, IL-8, IL-1β and TNF-α from airway epithelial cells or antigen presenting cells [54]. In vitro experiments demonstrated that PM_2.5_ exposure induced IL-8 gene expression by inducing oxidative stress and endocytosis in airway cells [46]. MX2 is a myxovirus resistance gene located in human chromosome 21q22.3, encoding MXB protein [55]. Recent studies have shown that MXB acts as a limiting factor for interferon induction and targets HIV-1 by binding to the HIV-1 capsid and inhibiting its nuclear import, thereby inhibiting replication at the postentry step [56]. This suggests that MXB proteins are involved in the innate immune response associated with viral infection and tumor development. Inhalation of moxa smoke can increase the degree of inflammatory cell infiltration and the expression of MX2 in the lung tissue of rats [57]. The expression of MX2 is associated with COVID-19 [58]. However, the toxicological mechanism of MX2 and PM_2.5_ has not been clearly reported, and we searched the CTD database for MX2 as a possible biomarker of PM_2.5_. Interleukin-1 (IL-1) is one of the major proinflammatory cytokines secreted by adipose tissue [59]. Interleukin-1A (IL-1A, IL-1α), a member of the IL-1 family, is involved in immune response, inflammatory response and tumorigenesis [60]. Several studies have shown that IL-1A expression is higher in a variety of tumor tissues than in paracancerous tissues [61]. Lipid synthesis was significantly increased in SZ95 sebaceous cells exposed to low concentrations of PM_2.5_, while increased PM_2.5_ concentration rapidly reduced lipid synthesis and stimulated the release of pro-inflammatory cytokines (such as IL-1α, IL-6 and IL-8) to participate in the inflammatory environment and the pathogenesis of various sebaceous gland related diseases [47]. Prostaglandin-endoperoxide synthases (also called COX) are rate-limiting enzymes for the conversion of arachidonic acid (a product of damaged cell membranes) to prostaglandins, which are present in at least two isoforms, PTGS1 (COX-1) and PTGS2 (COX-2). PTGS2 expression is induced by cytokines and growth factors and is upregulated during inflammation [62]. PM_2.5_ induces an inflammatory response in macrophages by activating TLR4/NF-κB/COX-2 signaling pathway [63]. Even limited PM_2.5_ exposure increased the expression of the pro-inflammatory molecular marker COX-2 in microglia [64]. When A549 cells are exposed to Cooking oil fumes (COFs) -PM_2.5_, the expression of COX2 is increased, which is involved in cell inflammation and apoptosis [65].

**Table 1:** The Expression of Hub Genes in PM_2.5_ by Literature Search

**Gene**	**Express**	**Literature Search**	

		**Sample**	**Conclusion**
JUN	Upregulation	NHEKs and RPE cells	PM_2.5_ transcriptionally upregulated small proline rich protein 3 (SPRR3) expression by activating c-Jun, and ectopic expression of SPRR3 inhibits suppressed the ciliogenesis.
CXCL8	Upregulation	BEAS-2B and THP-1 cells	Exposure to PM_2.5_ induces CXCL8 gene expression through oxidative stress induction and endocytosis in airway cells.
IL1A	Upregulation	16-HBE cells	The induction of AHR signaling by PM_2.5_ and IL-1-driven epithelial inflammation lead to changes in mucus secretion and dysfunction of ciliated cells.
PTGS2	Upregulation	PBMC	PTGS2 levels decreased after long-term exposure to high concentrations of air pollutants.
MX2	Downregulation	-​-​-​-​-​-​-​-​-​-​-​-​-​-	

We used CIBERSORT to evaluate the degree of infiltrating immune cells in this study. Dendritic cells (DCS) are a heterogeneous population of cells that are present in most peripheral tissues in small numbers and take up antigens through phagocytosis, macropinocytosis, and receptor-mediated endocytosis [66]. Although there is no significant difference in the changes of immune cells after BEAS-2B cells exposure to PM_2.5_, there is a decreasing trend of Dendritic cells resting, which may be involved in immune regulation. We found a negative correlation between resting monocytes and Dendritic cells resting. Airborne particulate matter decreased the uptake of virus by alveolar macrophages and inhibited the function of dendritic cells [67]. PM_2.5_ exposure can induce monocytes and neutrophils to participate in inflammatory response [68].

Notwithstanding strengths of bioinformatics analysis, including differential expression analysis, function analysis, pathway analysis and immune cell infiltration analysis, certain limitations merit consideration. In this study, a dataset was selected to explore the mechanistic effects of PM_2.5_ in BEAS-2B cells, but we avoided dataset quality issues by combining CTD data, literature review for validation of five hub genes. What’s more, lacking of experimental validation of hub genes is a limitation of the study. We will next verify hub genes mechanism through in vitro experiments. Bioinformatics analysis of toxicological relationships can effectively screen genes involved in toxicological molecular regulation. The effects of the PM_2.5_ on the hub genes expression in BEAS-2B cells identified in this study will provide evidence/basis for further validation using rigorous design and molecular techniques.

## 5. Conclusions

In summary, five hub genes and multiple pathways identified in this study may be related to the molecular mechanism of PM_2.5_. We speculate that these candidate genes or pathways may play different roles in the development of BEAS-2B cells exposed to PM_2.5_. These key genes and pathways deserve more exploration and validation in the future.

## Abbreviations

PM_2.5_: Particulate matter 2.5
DEGs: Differentially expressed genes
GO: Gene Ontology
KEGG: Kyoto Encyclopedia of Genes and Genomes
PPI: protein-protein interaction
BP: biological processes
CC: cell components
MF: molecular functions
CTD: Comparative Toxicogenomics Database
ROS: reactive oxygen species

## Supplementary information

**Additional file 1:** **Figure 1S.** Standardized comparison of significant DEGs. (A) Boxplot of DEGs before normalization. (B) Standardized boxplot. **Figure 2S.** The chord diagram of DEGs GO analysis. **Figure 3S.** PPI network analysis for 53 DEGs. **Table 1S** Top ten hub genes obtained by five algorithms of Cytohubba. **Table 2S** Mineralogical composition of PM_2.5_. **Table 3S.** Concentrations of PAHs (µg/kg) in PM_2.5_ from biomass combustion.

## Declarations

### Availability of data and materials

Data from the GEO database, please visit: https://www.ncbi.nlm.nih.gov/geo.
