# Supplementary material for: Identification of differentially expressed genes and pathways in BEAS-2B cells upon long-term exposure to particulate matter (PM2.5) from biomass combustion using bioinformatics analysis
Source: Environ Health Prev Med. 2023 Sep 15;28:51. doi: 10.1265/ehpm.22-00272 (PMC10519835; doi:10.1265/ehpm.22-00272)
Supplement: Supplementary file 1 — Additional file 1: Figure 1S. Standardized comparison of significant DEGs. (A) Boxplot of DEGs before normalization. (B) Standardized boxplot. Figure 2S. The chord diagram of DEGs GO analysis. Figure 3S. PPI network analysis for 53 DEGs. Table 1S Top ten hub genes obtained by five algorithms of Cytohubba. Table 2S Mineralogical composition of PM2.5. Table 3S. Concentrations of PAHs (µg/kg) in PM2.5 from biomass combustion. [file ehpm-28-051-s001.docx]

Supplementary materials

Figure 1S. Standardized comparison of significant DEGs. (A) Boxplot of DEGs before normalization. (B) Standardized boxplot


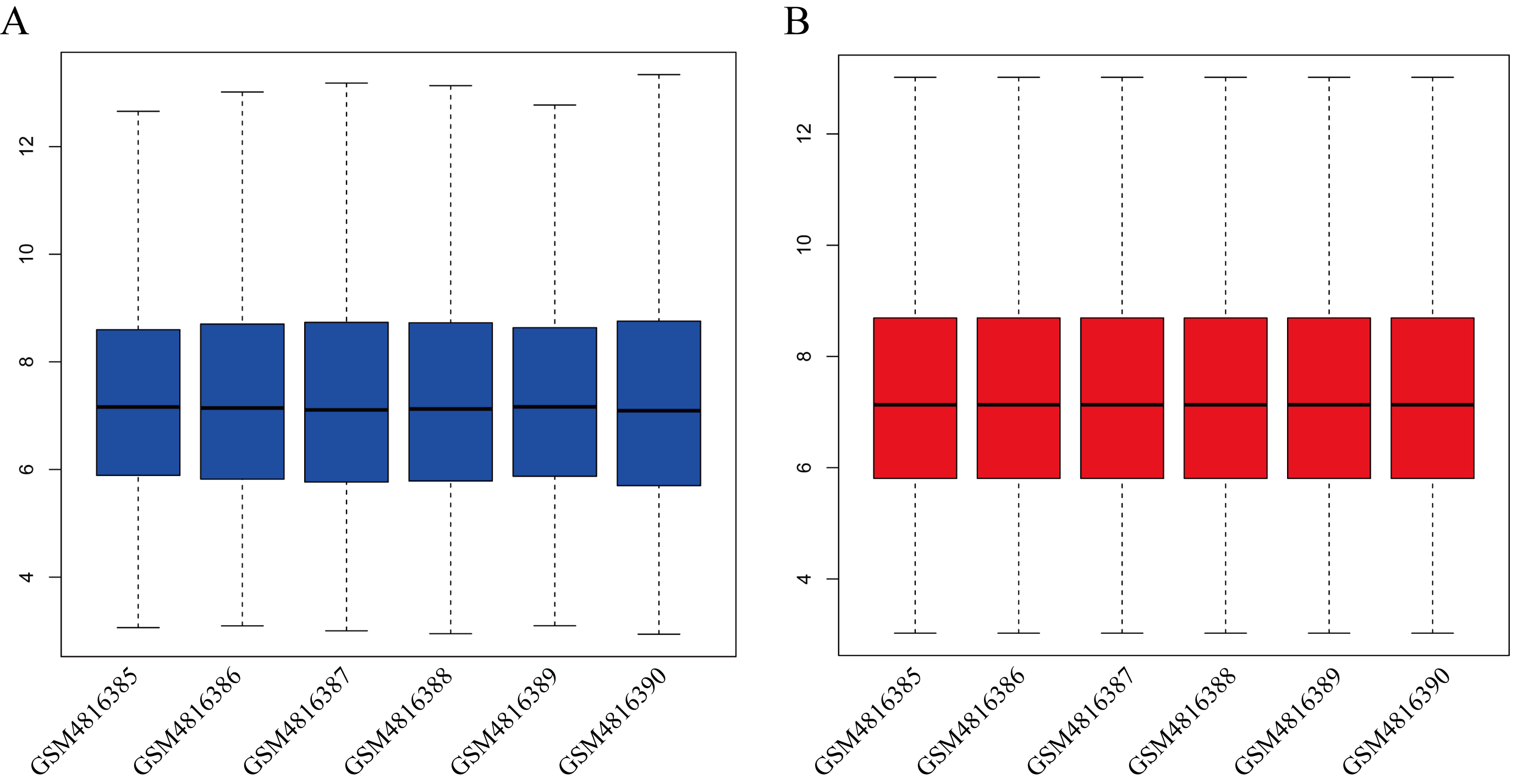


Figure 2S. The chord diagram of DEGs GO analysis

Figure 3S. PPI network analysis for 53 DEGs.


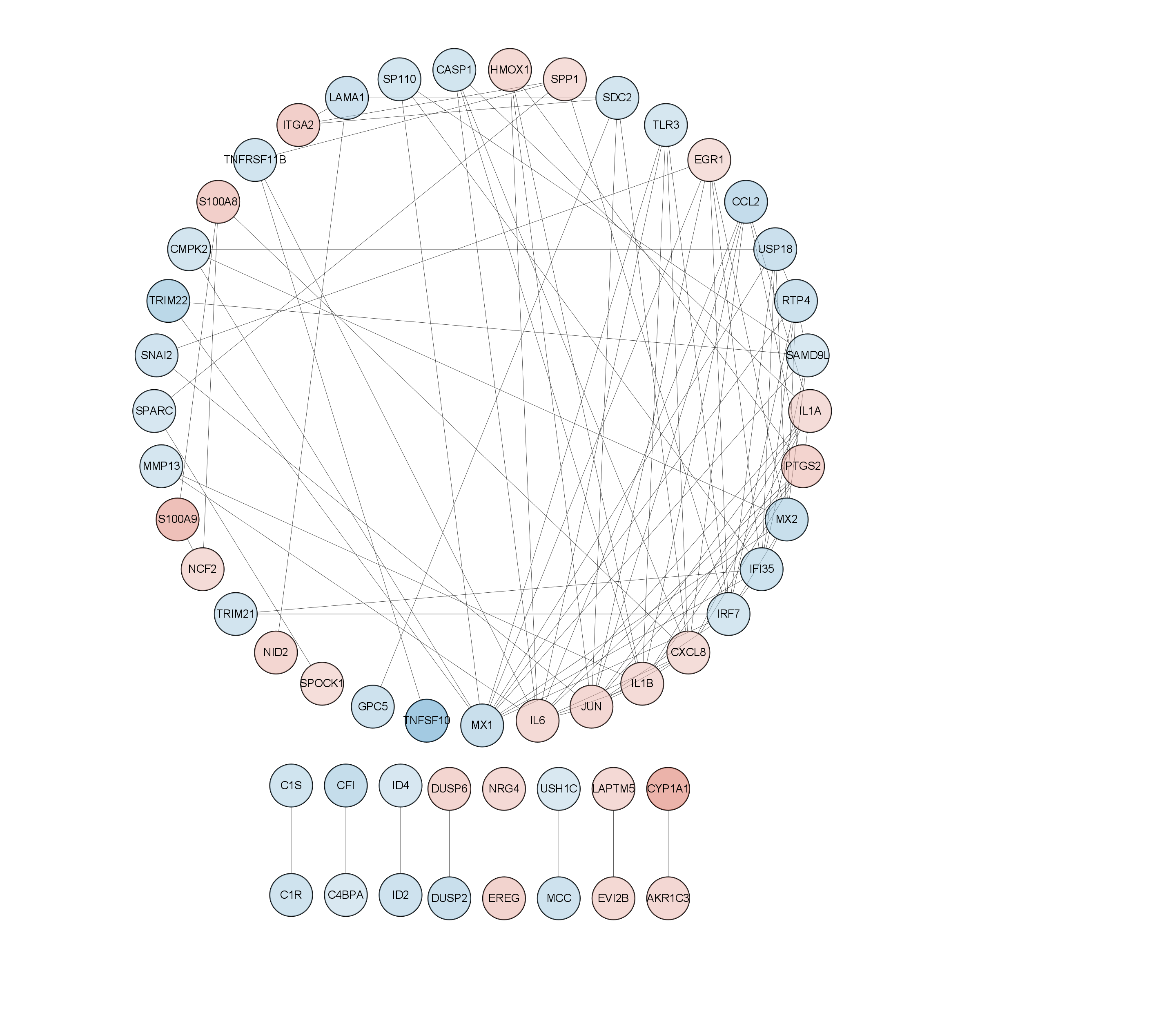


Table 1S Top ten hub genes obtained by five algorithms of Cytohubba.

| MCC | DMNC | MNC | Degree | EPC |
| --- | --- | --- | --- | --- |
| IL6 | CCL2 | MX1 | IL6 | JUN |
| IL1B | IL1A | IL1B | JUN | IL6 |
| CXCL8 | PTGS2 | IL6 | MX1 | IL1B |
| JUN | RTP4 | IFI35 | IRF7 | CXCL8 |
| IL1A | USP18 | IRF7 | IL1B | IRF7 |
| PTGS2 | HMOX1 | CXCL8 | CXCL8 | MX1 |
| CCL2 | CASP1 | JUN | IFI35 | IFI35 |
| MX1 | JUN | MX2 | MX2 | PTGS2 |
| IFI35 | MX2 | IL1A | IL1A | IL1A |
| MX2 | CXCL8 | PTGS2 | PTGS2 | MX2 |

| Component | percentage portion |
| --- | --- |
| Arcanite (K_2_SO_4_) | 47% |
| Amorphous | 30% |
| Sylvite (KCl) | 5% |
| Larnite (Ca_2_SO_4_) | 5% |
| Thenardite (Na_2_SO_4_) | 2% |
| Merwinite (Ca_3_Mg(SiO_4_)_2_) | 2% |
| Periclase (MgO) | 2% |
| Portlandite (Ca(OH)_2_) | 2% |
| Aragonite (CaCO_3_) | 2% |
| Syngenite (K_2_Ca(SO_4_)_2_ ∙ H_2_O | 1% |
| Dolomite (CaMg(CO_3_)_2_) | 1% |
| Lime (CaO) | 1% |

Table 2S Mineralogical composition of PM2.5

Table 3S. Concentrations of PAHs (µg/kg) in PM2.5 from biomass combustion

| PAH | µg/kg | PAH | µg/kg |
| --- | --- | --- | --- |
| Fluoranthene | 50.67 | Dibenzo[a,e]pyrene | 1.72 |
| Pyrene | 41.98 | 2-Methylchrysene | 1.31 |
| Phenanthrene | 36.01 | 1-Methylfluorene | 1.24 |
| Retene | 33.43 | Naphtho[1,2-b]fluoranthene and Naptho[2,3-a]pyrene | 0.93 |
| Naphthalene | 22.57 | 6-Methylbenzo[a]anthracene | 0.84 |
| Chrysene | 15.27 | Perylene | 0.73 |
| 1-Methylpyrene | 14.22 | Naphtho[2,3-b]fluoranthene | 0.59 |
| Benzo[e]pyrene | 12.27 | Dibenzo[a,l]pyrene | 0.49 |
| Benzo[b]fluoranthene | 12.23 | Dibenzo[a,i]pyrene | 0.38 |
| 9-Methylphenanthrene | 11.52 | 9-Methylanthracene | 0.23 |
| 2-Methylnaphthalene | 11.44 | 1,8-Dimethylnaphthalene | 0.17 |
| 2,6-Dimethylnaphthalene | 10.63 | 6-Methylchrysene | 0.12 |
| Benzo[ghi]fluoranthene | 10.15 | Anthanthrene | <2.50 |
| Anthracene | 7.38 | 3-Methylcholanthrene | <1.25 |
| 1-Methylphenanthrene | 7.3 | 6-Methylbenzo[a]pyrene | <1.25 |
| Benzo[a]anthracene | 6.7 | 9,10-Dimethylanthracene | <0.50 |
| Triphenylene | 6.41 | 5-Methylbenzo[a]anthracene | <0.50 |
| Benzo[a]pyrene | 5.29 | 4-Methylchrysene | <0.50 |
| Fluorene | 4.67 | Dibenzo[a,h]pyrene | <0.50 |
| Indeno[1,2,3-cd]pyrene | 3.86 | Naphtho[1,2-k]fluoranthene | <0.38 |
| Acenaphthene | 3.64 | Dibenzo[a,e]fluoranthene | <0.38 |
| Benzo[k]fluoranthene | 2.74 | Naphtho[2,3-k]fluoranthene | <0.38 |
| Benzo[ghi]perylene | 2.42 | Naphtho[2,3-e]pyrene | <0.38 |
| 7,12-Dimethylbenzo[a]anthracene | 2.27 | Dibenzo[a,j]anthracene | <0.25 |
| Coronene | 2.1 | Dibenzo[a,c]anthracene | <0.25 |
| Benzo[c]phenanthrene | 1.93 | Dibenzo[a,h]anthracene | <0.25 |
| 7H-Benzo[c]fluorene | 1.73 |  |  |
